# Advances in animal models of obstructive sleep apnea

**DOI:** 10.3389/fmed.2023.988752

**Published:** 2023-02-07

**Authors:** Shimin Zong, Peiyu Du, Hejie Li, Miao Wang, Hongjun Xiao

**Affiliations:** Department of Otorhinolaryngology, Union Hospital, Tongji Medical College, Huazhong University of Science and Technology, Wuhan, China

**Keywords:** obstructive sleep apnea, animal model, chronic intermittent hypoxia, spontaneous upper airway stenosis, brachycephalic obstructive airway syndrome, polysomnography

## Abstract

Animal experiments play an important role in the study of the pathogenesis of human diseases and new methods of diagnosis and treatment. Due to the great differences in the anatomical structure and physiology of the upper airway between animals and humans, there is currently no animal model that can fully simulate the pathological anatomy and pathophysiological characteristics of human obstructive sleep apnea (OSA) patients. Herein, we summarizes the construction methods of several OSA animal models that have been widely used in the studies published in the last 5 years, the advantages and limitations of each model as well as related evaluation techniques are described. This information has potential to provide further guide for the development of OSA related animal experiments.

## 1. Introduction

Obstructive sleep apnea (OSA) is a sleep breathing disorder characterized by sleep snoring with apnea and daytime sleepiness as the main clinical manifestations, which can lead to multiorgan and multisystem damage such as hypertension, coronary heart disease, arrhythmia, cerebrovascular disease, cognitive impairment and type 2 diabetes. The main pathophysiological features of OSA including upper airway stenosis, upper airway collapse during sleep, intermittent hypoxia, hypercapnia, and sleep structure disturbance (1).

Studies on obstructive sleep apnea are mostly clinical studies so far. Although clinical studies has advantage in obtaining a relatively high-level evidence-based medical evidence, the cost of time and manpower is huge. Individual differences of subjects, compliance, and ethical risks, meanwhile, are unavoidable. Animal experiments can avoid these problems and mainly show the following advantages: (1) animal disease models greatly shorten the research period, especially studies on the long-term efficacy or effects of treatments; (2) the application of inbred lines, closed groups, and genetically engineered animals improves the homogeneity of experimental animals. Therefore, the complex problems in the human body can be simplified and various factors can be explored in detail; (3) New drugs, invasive or potentially harmful interventions may not enter clinical studies until their safety has been confirmed in animal studies; (4) animal experiments are easier to obtain a variety of tissue samples for further cellular, molecular, gene and other levels of research. Therefore, it is necessary to construct an animal model to simulate the pathophysiological characteristics of OSA patients.

Due to the significant differences between the upper airway anatomy of animals, especially non-primates, and humans, no animal were found to spontaneously acquired OSA. Therefore, artificial intervention to make animals possess part of pathophysiological characteristics of OSA patients is a more widely used modeling idea. This review summarizes the representative animal models of OSA in the past 5 years and analyzes the advantages and disadvantages of each model, in order to provide help for the further development of OSA-related animal experiments.

## 2. Characteristics, advantages, and limitations of existing OSA animal models

### 2.1. Chronic intermittent hypoxia (CIH) models

These models simulate the pathophysiological process of intermittent hypoxia and damage to related organ systems in OSA patients through chronic intermittent hypoxia in animals. There were two approaches to build this model.

In the first model, animals were intubated under general anesthesia and ventilated with a ventilator. The process of hypoxia was simulated by periodic closure of the tracheal tube (complete or partial closure), and the animal was immediately restored to ventilation after closure. Each anoxic-ventilation cycle was counted as one cycle, and the number of anoxic-ventilation cycles per hour was identified as the apnea hypopnea index (AHI). The simulation of different AHI parameter could be achieved by adjusting the hypoxia and/or ventilation time. The severity of OSA that needed to be simulated determined the total duration of intermittent hypoxia measured each day and the total number of days of the experiment. This model has been used to detect the characteristics of CIH-related multiorgan and multisystem damage and explore the corresponding potential interventions (2–4). The establishment of this model requires daily tracheal intubation under general anesthesia for animals, which is relatively complex, and experimental animals need to bear the risk of general anesthesia repeatedly. Therefore, the modeling operation itself has certain potential safety risks for animals.

**FIGURE 1:**
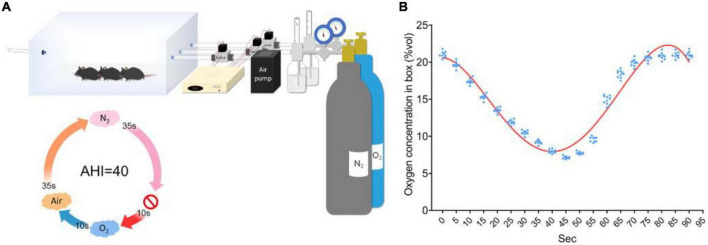
Diagram of the CIH animal model established by a high/low oxygen animal culture system with an adjustable oxygen concentration. **(A)** Schematic of the OSA induction protocol. **(B)** Oxygen concentration was monitored in real time over 90-s cycles (27).

The other CIH model does not require anesthesia. The core of this model is a specially designed laboratory cage (e.g., the Biospherix high/low oxygen animal culture system) that can adjust the composition of the internal gas. The device pumps air, oxygen or nitrogen into the cage according to the required proportion through an air pump to achieve accurate regulation of oxygen concentration in the cage. The animals could drink and eat freely in the cage, and the humidity and pressure in the cage were maintained as the same level as those in the daily environment. During the modeling process, the cage was filled with nitrogen to reduce the oxygen concentration so that the animals were in an anoxic environment, and then the cage was filled with oxygen or air to simulate the resumption of ventilation of the animals. Similar to the occluding endotracheal intubation model under general anesthesia, this model could simulate different degree of AHI by setting the duration and cycle of hypoxia ventilation and simulate the duration of OSA patients by setting the duration of the experiment (5–28). A typical molding process is shown in Figure 1.

This model does not require anesthesia, the gas concentration can be accurately controlled, and the modeling process is safe, stable and reproducible. It is especially suitable for small animals with a high risk of anesthesia or difficult tracheal intubation. It is the most widely used OSA animal model at present. However, this model can only simulate the pathophysiological changes caused by human OSA-related CIH, there is no upper airway stenosis or collapse similar to that in OSA patients. There is no conclusive evidence of neuromuscular excitability decrease and sleep structure disorder in animals in this model. Specifically, this model should be called the CIH model rather than the OSA model (29). On the basis of these models, if a video detection system is applied, and the sleep of animals can be disturbed by moderate physical stimuli such as sound and vibration when oxygen concentration reaches a trough during sleep, to realize the simulation of arousal, will the degree of simulation of OSA patient pathophysiological characteristics of this animal model be improved? This deserves further consideration. In addition, is the detection of SpO_2_ in animals consistent with trends in environmental oxygen concentration? To what extent can this model simulate the variation of SpO_2_ curve in OSA patients during sleep? It is also an issue worth thinking about. Since there is no animal upper airway stenosis in this model, it is not suitable for the study of OSA surgery-related problems.

### 2.2. Model of upper airway collapse induced by a negative pressure environment

It has been suggested that the upper airway during sleep normally acts like a collapsible catheter and that its patency depends on the transmural pressure—the difference between the pressure in the airway lumen and the pressure exerted by the tissues surrounding the collapsed site. In healthy people, the transmural pharyngeal pressure is maintained from 0 to +10 mmHg during sleep. Upper airway obstruction in OSA patients is associated with transmural negative pressure during sleep (30). King et al. found that when healthy controls were exposed to an environment of -10 cm H_2_O to sleep, polysomnography (PSG) results showed that upper respiratory tract obstruction did occur during sleep and induce OSA (31). The results provide strong support for the rationality of the above views.

Based on the above theory, Liu et al. placed a 6-month-old mini-pig weighing 17 ± 1.5 kg in a 1.5 m × 2 m × 3 m metal chamber with a foam interlayer. The pressure in the chamber was maintained at 0.96 ± 0.01 kPa, and the oxygen concentration was maintained at 22.5 ± 0.3%. Animals were exposed to this low-pressure environment 12 h per day for 6 months. Animal behavior, such as activity, sleep, snoring and other indicators were observed and analyzed to evaluate the similarity between the model and human OSA pathophysiological characteristics. The results showed that, after 6 months of feeding in negative pressure environment, there were no significant differences in body weight and food intake compared with the control group. But meanwhile the experimental group showed activity decreased, sleep time increased, snoring intensity increased (43–51 dB), respiratory rate increased, peak value of negative respiratory pressure increased (3.5–4 kPa), and respiratory pressure waveform became irregular. The above characteristics are partly consistent with the parameter characteristics of human OSA patients (30).

Compared with the CIH model, this model has the theoretical basis of upper airway collapse, which may be more consistent with the pathophysiological characteristics of human OSA. If the experimental animals in this model can be examined by electronic laryngoscopy under negative pressure to obtain direct evidence of upper airway collapse and stenosis, the model will be more convincing. Since the circulatory system of pigs is similar to that of humans in evolution, this model is particularly suitable for the study of cardiovascular diseases caused by OSA.

### 2.3. Spontaneous upper airway narrowing model

It is a feasible idea to establish an OSA animal model by injecting dilatants (such as polytetrafluoroethylene and hydrophilic polyacrylamide gel) into soft tissues of the upper airway, such as the tongue or the soft palate, which can cause persistent swelling and narrowing of the upper airway soft tissues (32–37). Polytetrafluoroethylene (PTFE) is an inert swelling agent used in the treatment of vesicoureteral reflux. Lebek et al. injected 35 μm PTFE (50 mg PTFE dissolved in 100 μL glycerol) into the tongue root of 8- to 12-week-old male C57BL/6 mice by multipoint injection. After injection, there was no significant increase in body weight of the mice, but some typical signs of OSA appeared, such as upper airway narrowing caused by significantly increased tongue volume. The frequency of inspiratory effort and apnea increased significantly in mice (34). Similarly, Lu et al. injected 2 ml hydrophilic polyacrylamide gel into the submucosal muscular layer of the soft palate 1.5 cm from the posterior edge of the hard palate in New Zealand rabbits. After 8 weeks, the OSA group showed progressively worsening sleep apnea and hypopnea, as well as superficial cyanosis during sleep, leakage of urine after waking was also found in OSA group. Upper airway CT showed that the sagittal diameter cross section of the upper airway decreased in the OSA group. Results of the PSG indicated that sleep breathing disorders were present in OSA group, nasal airflow decreased with the increase in breathing effort, SpO_2_ decreased by more than 8% of the baseline value, and the AHI in the OSA group was significantly higher than that in the control group (OSA group: 30.77 ± 2.30, control group: 2.78 ± 0.27). In this model, the authors also found that mandibular advancement devices (MADs) can be effective in correcting OSA (33, 37).

The above model of spontaneous upper airway narrowing is relatively simple to operate and has the advantage of simulate the anatomical and pathophysiological characteristics of upper airway obstruction in OSA patients. Meanwhile, the effect of modeling and intervention can be evaluated through medical imageology and PSG. Hence, the results are even more convincing. Some researchers believe that rabbits can be trained to acquire the habit of supine sleep, and PSG can be performed during sleep, which can better simulate the characteristics of OSA patients (37). Theoretically, in addition to the pathophysiological changes related to CIH, there should also be changes in sleep structure disorder and decreased neuromuscular excitability. In the future, it is feasible and necessary to apply the model to further study the above content. One problem of the model is that animals may die from massive bleeding at the injection site, uncontrolled tissue edema or severe infections (34). Increasing the sample size and standardizing fine operations may be effective methods to avoid the occurrence of the above events.

### 2.4. Brachycephalic obstructive airway syndrome (BOAS)

In the process of natural evolution, dogs do not show the anatomical changes and pathophysiological characteristics of upper airway similar to those of human OSA patients. However, dogs have been artificially bred by humans for their own aesthetic needs, such as bulldogs, pugs, Pekingese and Shih Tzu, have a more extreme look—a large, flat, round face. These dogs are characterized by abnormal facial structure, hypoplasia of the upper airway, laboring for breath, pig-like breathing, snoring, chronic cough, poor physical strength or endurance, heatstroke, fainting due to hypoxia, and even sudden death. This condition is called brachycephalic airway syndrome (BOAS). Dogs with BOAS usually have the following pathological features: (1) nasal collapse: the soft tissue of the nasal cavity collapses inward during the inhalation phase, resulting in nasal narrowing; (2) the soft palate is too long, extending to the end of the airway, causing pharyngeal cavity stenosis; and (3) laryngeal vesicle eversion: the laryngeal sac (unique to dogs, laryngeal and peritracheal soft tissue) is pulled into the trachea during the inspiratory phase, resulting in upper airway obstruction (38). The above characteristics are highly similar to the anatomical and pathophysiological characteristics of OSA patients, so it is considered as a natural animal model of OSA.

For dogs with BOAS, animal doctors usually adopt the following treatment methods: (1) control body weight and avoid increasing oxygen load; (2) soft palate resection: excision of the long soft palate under general anesthesia to expand the pharynx cavity; (3) laryngectomy: usually performed at the same time as soft palatectomy; and (4) rhinoplasty: removal of the excess tissue in the nasal cavity and suturing of the wound to enlarge the nasal cavity. After surgery, upper airway obstruction due to bleeding and local tissue edema was the most serious complication, thus should be closely monitored. The above therapeutic methods are similar to those in human OSA therapy. On the one hand, this model simulates the characteristics of human OSA upper airway collapse, and has the characteristics of upper airway stenosis in different anatomical planes. On the other hand, the above mentioned surgical method is relatively mature in veterinary medicine with strong security. Based on the analysis above, this model is currently a rare animal model that can be applied to studies on human OSA-related surgery and perioperative management (39, 40). For example, partial soft palate resection in dogs can be performed by an ultrasonic knife or laser, and the hemostasis of the soft palate margin is highly similar to human OSA surgery. Therefore, this model is quite suitable for evaluating the safety and effectiveness of new hemostatic materials, new suture materials or surgical techniques in human OSA surgery.

However, this model also has the following limitations: (1) The genetic background of experimental dogs is complex, it is difficult to achieve homogeneity, and the degree of maxillofacial-upper airway structure malformation is different in each short-beamed dog. In addition, due to the different grade of maxillofacial-upper airway malformation, each short-beamed dog may have one or more types of OSA, and the severity of OSA also varies. In view of the above individual differences, it is worth further trying to establish a closed group by breeding dogs with typical BOAS symptoms to breed relatively homogeneous OSA model animals. (2) Compared with rodents, dogs have relatively poor reproductive ability and it is difficult to obtain a large number of experimental animals in a short time. (3) OSA symptoms in BOAS dogs are usually caused by abnormal maxillofacial development and are therefore poorly modeled for obesity-related OSA.

### 2.5. An obesity-related OSA animal model—New Zealand obese (NZO) mice

In a study published in 2011, Brennick et al. used respiratory gating MRI to compare the difference in cross-sectional area at the tail of the pharyngeal cavity during breathing between Obese New Zealand (NZO) mice (weight 50.4 ± 4.0 g) and New Zealand white (NZW) mice (weight 34.7 ± 1.3 g normal control). They found that (1) at 20–24 weeks of age, NZO mice developed excessive fat accumulation around the pharyngeal cavity, further leading to pharyngeal stenosis; (2) cross-sectional area of the posterior pharyngeal cavity: inspiratory phase > expiratory phase in NZO mice and expiratory phase > inspiratory phase in NZW mice. The cross-section of hypopharynx changes most obviously during respiratory movement (3) Obesity is correlated with the cross-sectional area of the upper airway during breathing, which is different from human OSA patients. In view of the above differences, the authors proposed the following hypothesis: the thoracic cavity of obese mice needs to produce more negative pressure during the inspiratory phase and is more prone to a decrease in the neuromuscular excitability of the pharynx. In rodents, only the wall of the posterior pharyngeal cavity is composed of soft tissue, and the rest of the pharyngeal wall is attached to the bony structures, while the patency of the airway at the posterior pharyngeal cavity depends on the mechanical expansion of the thoracic cavity and the non-bony pharyngeal wall during inspiration. Therefore, the pharyngeal cross-area in the inspiratory phase of NZO mice increases (41).

Baum et al. in 2018 conducted a comparative study between modified NZO mice–New Zealand obese (NZO/HlLtJ) mice (weight 51.7 ± 0.79 g) and New Zealand black (NZB/BlNJ) non-obese control mice (weight 32.31 ± 1.05 g) using small animal PSG. The results showed that, in both active and rest periods, NZO mice showed more frequent apnea, hypopnea, and even sleep under standing posture, frequent SpO_2_ decrease (NZO: 95.3 ± 0.2%, 92.5% < SpO_2_ < 97.3%, NZB: SpO_2_: 98.9 ± 0.1%, 98.0% < SpO_2_ < 99.4%), and the time of arousal increased significantly. During the rest period, the NZO mice showed hyperventilation, which was manifested by increased breathing rate and shortened time of inspiration phase. However, during the activity period, the sleep time of the NZO mice was longer than that of the NZB mice, while the activity time and intensity were weaker than that of the NZB mice, indicating that daytime sleepiness existed in NZO mice during the activity period, which was similar to human OSA patients (42).

Obstructive sleep apnea is a complex disease involving anatomical, neural, metabolic and other abnormalities. However, traditional OSA animal models can only imitate a certain aspect of human OSA pathophysiology. It is rare that NZO mice have multiple pathophysiological characteristics at the same time: apnea/hypopnea, fragmented sleep and daytime sleepiness. Of the existing animal OSA models, it is the most similar to human OSA in terms of pathophysiology (42). Some researchers have pointed out that the degree to which an animal model can simulate human disease (i.e., the application value of the model in research) depends on three conditions: (1) homology, (2) isomorphism with the pathophysiology of the human disease, and (3) predictability. The NZO MICE model has both condition (1) and condition (2) (43).

The advantages and disadvantages of the above models are listed in a visual way in the Table 1.

**TABLE 1:** The advantages and disadvantages of the above models are listed.

OSA animal models		Advantages	Disadvantages
Chronic intermittent hypoxia (CIH) models	General anesthetic tracheal intubation	Precise control of periodic hypoxia; No surgery required	Higher risk of general anesthesia; only simulates periodic hypoxia without upper airway stenosis
	Laboratory cages	No anesthesia required; precise control of periodic hypoxia	Simulates only periodic hypoxia without upper airway stenosis
Model of upper airway collapse induced by a negative pressure environment		More consistent with the pathophysiological characteristics of human OSA	Lack of objective evidence such as laryngoscopy or sleep records
Spontaneous upper airway narrowing model		More consistent with the pathophysiological characteristics of human OSA;	Risk of bleeding, uncontrolled tissue oedema or serious infection at the injection site; lack of evidence of sleep records
Brachycephalic obstructive airway syndrome (BOAS)		More consistent with the pathophysiological characteristics of human OSA; Proven surgical methods; High level of security	Difficult to obtain homogenous laboratory animals; relatively poor canine reproduction; poor mimicry of obesity-related OSA
New Zealand obese (NZO) mice		More consistent with the pathophysiological characteristics of human OSA; High level of security; No surgery required	(–)

## 3. Key evaluation techniques for OSA animal models

Polysomnography is the reference standard for diagnosing OSA in humans. Electronic nasolaryngoscopy and upper airway imaging are common methods for the analysis and diagnosis of upper airway obstruction plane, and these diagnostic techniques are also critical to evaluate the efficacy of OSA animal models. However, the body size of experimental animals is usually small, their upper airway structure is also different from that of human beings, and it is difficult to control the activities and sleep under non-anesthesia. All of these problems challenges for the application of OSA diagnostic technology in living animals. With the continuous efforts and exploration of researchers all over the world, some diagnostic techniques have been successfully applied to OSA animal models.

### 3.1. PSG for small animals

**FIGURE 2:**
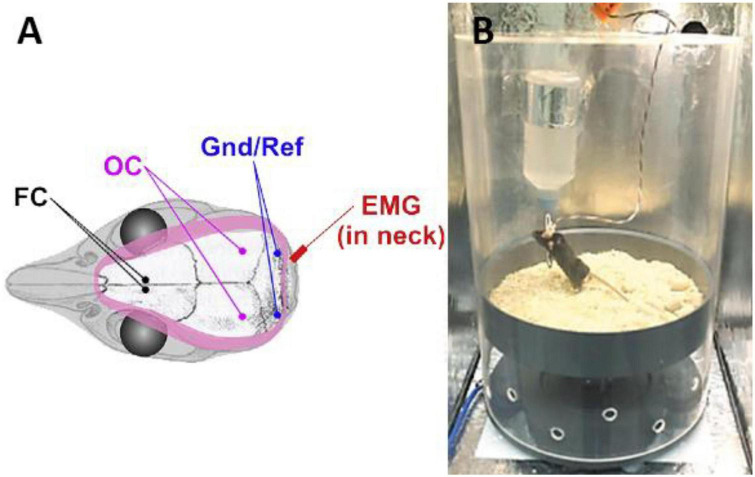
Schematic diagram of EEG/EMG electrode placement during PSG implementation in rodents **(A)** and the relationship between animals, wires and cages during data recording **(B)** (45, 46). FC, frontal cortex; OC, occipital cortex; Gnd/Ref, grounding/reference electrode; EMG, electromyography.

Polysomnography has been applied to evaluate a variety of OSA animal models, such as rabbits, pigs, dogs, rats and mice, among which it is difficult to implement PSG due to the small size of rodents (rats and mice), so PSG is the most representative. Manuel Silva-Perez et al. described the PSG procedure in small animals: (1) Electrodes were surgically fixed to the skull using miniature stainless steel screws. Each EEG electrode is largely wrapped in insulating material and surrounds multiple 1 mm long non-insulating tips. The electrodes were fixed to the parietal bone and encircled the bilateral cerebral hemispheres, the non-insulating tip touched the dura mater, and the reference electrode was located in the vermis of the cerebellum. (2) The EMG recording electrode was placed in the right acromion muscle of the neck. All electrodes are welded to an acrylic covered connector, with only the connector located outside the body. Anti-inflammatory therapy was performed for at least 1 week postoperatively. After the animal fully recovered from surgery, it was placed in a cage that could shield the electrical signal. The connector connects the signal amplifier to the signal recording device through a wire. It is emphasized that the wire does not restrict the normal activity of the animal. The data were recorded for 5 consecutive days, with the first 3 days as the adaptation period, and only the data of the second 2 days were used for analysis (44) (Figure 2).

Polysomnography performed by the above method can clearly distinguish sleep and wakefulness, such as rapid eye movement (REM), non-rapid eye movement (NREM), wakefulness and arousal. Combined with EMG information and respiratory amplitude and rhythm information obtained by the whole-body plethysmograph, it can highly simulate the data obtained by human PSG (34, 44–46), which basically satisfies the judgment and analysis of OSA-related pathophysiological processes in small animals.

### 3.2. Respiratory gating MRI

Brennick et al. applied respiratory gating MRI to the evaluation of upper airways in small animals and subsequent studies in 2009. This technique can be used to image the upper airway in small animals during supine breathing, which is closer to the natural sleeping position and can avoid breathing difficulties caused by impaired diaphragm function in anesthetized animals. Pressure-sensitive “pillow sensors” are used to monitor and record breathing during the examination. The pillow sensor is a non-invasive and easy-to-operate respiratory motion sensing device that can provide a reliable trigger signal to start respiratory gating MRI to obtain MRI scan images of the upper airway synchronized with respiratory motion (41, 47).

It should be pointed out that the above-mentioned PSG for small animals may not be suitable for medium-sized animals such as dogs and pigs, since these animals may rip away the wires while awake, and the monitoring of EEG and EMG under anesthesia is not convincing. Effective ways to induce natural sleep in these animals are still lacking. On the other hand, perhaps due to the upper airway diameter of animals (especially rodents) is small, there is currently no sufficiently slender endoscopic that are slim enough to observe their upper airway status. With the progress of endoscopy manufacturing technology, it is hoped that in the near future, there will be an electronic rhinolaryngoscopy system for small animals, which will bring positive assistance to the establishment of a more simulated OSA animal model. Rabbit is the only animal reported so far that can induce natural sleep, and there is no obvious difficulty in the implementation of PSG in rabbits (37), so rabbits may have unique advantages in the establishment of OSA animal models.

## 4. Discussion

Reliable animal models of OSA play an indispensable role in the study of the pathogenesis, diagnosis and treatment of OSA. However, due to the great differences in upper airway anatomical structure and physiology between animals and humans, it is unlikely to develop an animal model that can completely simulate the pathological anatomy and pathophysiological characteristics of human OSA patients.

According to clinical practice guidelines, the criteria for sleep fragmentation are EEG microarousals (≥ 3 s) at a frequency of 10 or more events per hour (48). Sleep fragmentation is one of the main features of OSA (49). Meanwhile, sleep fragmentation leads to hypertension, drowsiness and other symptoms caused by OSA, which is important for the assessment of OSA hazards (50, 51).

Therefore, the presence and extent of sleep fragmentation can only be confirmed by PSG. The performance and optimization of PSG in animals is necessary to detect sleep fragmentation in OSA models.

Animal PSG is not widely used due to difficulties with experimental animal cooperation and limitations from instrumentation. Therefore studies on sleep fragmentation in animal models of OSA are scarce. With the continuous development of animal PSG technology, it is believed that more data will be available in the future to support the understanding of sleep fragmentation in OSA model animals.

With the continuous investment of researchers and the progress of science and technology, OSA animal models are being continuously developed and improved, emerging animal models will increasingly be able to mimic the characteristics of human OSA. Current OSA animal models have their own characteristics separately. In specific studies, different animal models can be selected according to different research purposes, and the existing models can be improved according to specific research purposes. PSG, upper airway imaging and electronic nasolaryngoscopy in small animals are critical measures for evaluating the reliability of OSA animal models. With the deepening of research, related evaluation techniques will become more mature.

## Author contributions

SZ and PD wrote the manuscript. All authors contributed to the article and approved the submitted version.
